# Poor extraction efficiencies of polystyrene nano- and microplastics from biosolids and soil

**DOI:** 10.1371/journal.pone.0208009

**Published:** 2018-11-29

**Authors:** Zhan Wang, Stephen E. Taylor, Prabhakar Sharma, Markus Flury

**Affiliations:** 1 Department of Crop & Soil Sciences, Washington State University, Pullman, WA, United States of America; 2 Department of Crop & Soil Sciences, Washington State University, Puyallup, WA, United States of America; 3 College of Land and Environment, Shenyang Agricultural University, Shenyang, China; 4 Key Laboratory of Arable Land Conservation (Northeast China), Ministry of Agriculture, Shenyang, China; 5 National Engineering Laboratory for Efficient Utilization of Soil and Fertilizer Resources, Shenyang, China; 6 School of Ecology and Environment Studies, Nalanda University, Rajgir, Nalanda, Bihar, India; VIT University, INDIA

## Abstract

Extraction and quantification of nano- and microplastics from sediments and soils is challenging. Although no standard method has been established so far, flotation is commonly used to separate plastic from mineral material. The objective of this study was to test the efficiency of flotation for the extraction of nano- and microplastics from biosolids and soil. We spiked biosolids and soil samples with polystyrene nano- and microbeads (0.05, 1.0, 2.6, 4.8, and 100 *μ*m diameter). Different extraction methods (w/ and w/o H_2_O_2_ digestion) were tested, and plastic beads were separated from mineral particles by flotation in a ZnCl_2_ solution. Plastic particles were quantified by UV-Vis spectrometry and gravimetrically. While large beads (100 *μ*m) could be quantitatively extracted (∼100%) from both biosolids and soils, smaller beads had low extraction efficiencies (ranging from 5 to 80%, with an average of 20%). Except for the 100 *μ*m beads, oxidation with H_2_O_2_ negatively impacted the extraction efficiencies. For the soil, extraction with water only, followed by flotation in a ZnCl_2_ solution, resulted in relatively high extraction efficiencies (>75%) for beads larger than 1 *μ*m, but low efficiencies (<30%) for the 0.05 and 1.0 *μ*m beads. Our results indicate that while flotation generally works to separate plastic nano- and microbeads in a solution, the challenge is to quantitatively extract nano- and microbeads from a biosolids or soil matrix. Samples high in organic matter content require removal of the organic matter, but the common method of H_2_O_2_ oxidation leads to poor extraction efficiencies for nano- and microbeads.

## Introduction

As the production of plastics is increasing worldwide in different sectors, plastic pollution of terrestrial and aquatic ecosystems continues to increase. Besides large, macroscopic plastics, an emergent pollutant is plastic in the nano- and microsize fraction [1, 2]. Microplastic refers to plastic particles less than 1 mm or 5 mm in size [3–5], while nanoplastic refers to plastic particles less than 1 *μ*m in size [6]. Nano- and microplastics are introduced into the terrestrial environment through a variety of pathways [6, 7]. Nano- and microplastics consist of either primary plastic particles, i.e., plastics produced in that size fraction for cosmetic and other industrial purposes, or secondary plastics, i.e., breakdown products from larger-sized plastic material [7, 8]. While pollution of marine ecosystems with plastic is well documented [8–10], less is known about plastic pollution in terrestrial ecosystems [7, 11]. Particularly, little is known about pollution with nano- and microplastics [5, 6].

To gain a better understanding of the impacts of nano- and microplastics in terrestrial environments, we need to quantify the nano- and microplastics in biosolids and soil. However, there is no uniform protocol for quantification of nano- and microplastics in biosolids and soils, and consequently, it is difficult to compare results from different studies where different methods of quantification were used. The first challenge is to separate nano- and microplastics from organic matter. Sieving just can remove the macroscopic organic materials [12, 13], but does not separate nano- and microplastics from all organic matter. As organic matter and microplastics have similar densities, they cannot be readily fractionated by density separation [14–18]. A common method of separation is by oxidizing natural organic matter with H_2_O_2_ [16, 19–21]. Overnight or 24 h treatment with 30% or 35% H_2_O_2_ was found not to affect plastic particles [19, 22, 23]; however, longer treatments up to 7 days can affect plastics [16].

Acids have been recommended for extracting plastics from organisms, but it was reported that nitric and perchloric acid can also degrade plastics, thereby affecting extraction efficiencies and plastic identification [24]. Alkaline [24, 25] and enzymatic digestion [22, 25] have been proposed as alternative to acid treatments; however, alkaline digestion has been found to degrade some plastics [23]. Cole and co-workers [25] compared the extraction efficiencies of acidic (HCl), alkaline (NaOH), and enzymatic (Proteinase-K) digestions for quantifying microplastics from marine samples containing plankton and found that the Proteinase-K digestion was the most successful to remove the biological material from the samples without affecting the microplastics. (Note: In a recent study published after we completed our work, different digestion methods were compared, i.e., H_2_O_2_, Fenton’s reagent, NaOH, and KOH, and Fenton’s reagent was found to be the optimal method to remove organic matter while not affecting plastics [23]).

The second challenge involves the separation of nano- and microplastics from mineral particles. The most common method to separate plastics from soil or sediment particles is flotation. Different types of solutions have been used to facilitate the separation during flotation, such as distilled water [26, 27], saturated NaCl (density ∼1.2 kg/L) [14, 15, 17, 18, 28], concentrated ZnCl_2_ (density ∼1.6–1.8 kg/L) [13, 20, 29], and concentrated NaI (density ∼1.6–1.8 kg/L) [3, 16, 21]. Among these solutions, ZnCl_2_ is recommended over NaI because it is less expensive, but shows higher extraction efficiency than NaCl [30].

Flotation has been used in different variations: (1) with a stagnant fluid, where particles are separated by density differences only [2, 11, 13, 14], (2) by froth flotation, where particles are separated by density and air bubbles, taking advantage of hydrophobicity differences [11, 20, 21], and (3) by hydrodynamic flotation where particle separation by density is aided by a hydrodynamic flow [16]. Flotation can be carried out under normal gravity or aided by centrifugation. No difference in extraction efficiencies between gravity and centrifugation separation was observed for plastic particles >500 *μ*m [11].

An alternative method based on the oleophilic properties of plastics has been proposed to extract microplastics with canola oil instead of density fractionation with a high-density fluid [31]. Another promising method based on pressurized fluid extraction of samples with methanol and dichloromethane has been reported [32]. This method provided high extraction efficiencies for plastic particles with mean diameter of 50 *μ*m from biosolids and soil samples. In principle, this method should work also for smaller particles in the lower micrometer and nanometer size range. However, it requires a special fluid extraction apparatus. Flotation methods, on the other hand, are more common and simpler to use, and have provided also high extraction efficiencies for particles in the greater than 50 *μ*m range.

Plastics separated by flotation methods usually range between 40 to 5000 *μ*m [15, 20]. There are no reports available about extraction of plastics in the lower micrometer (<40 *μ*m) and nanometer size range. These plastics may be difficult to extract due to their small size and also due to the longer time required for separation by gravity flotation.

The objective of this study was to test the flotation-based extraction method to quantify nano- and microplastics in biosolids and soils. We used polystyrene nano- and microspheres as model particles and extracted the particles from biosolids and soil samples. We used centrifugal and gravity flotation in combination with oxidation of organic matter to separate plastic particles from biosolids and soil. Particles were quantified spectrometrically and gravimetrically, and extraction efficiencies were determined.

## Materials and methods

### Plastic, biosolids, and soil materials

Spherical polystyrene beads (diameter 0.05, 1.0, 2.6, 4.8, and 100 *μ*m) were used as model nano- and microplastic particles (Table 1). Although most plastic particles in the environment are not spherical, we use spherical particles as a model, so that we can better compare experimental results with theoretical calculations. Biosolids samples were collected from the South Plant Waste Water Treatment Facility in Renton (WA) on Oct 7, 2016. These biosolids were the dewatered product of anaerobic digestion of solids separated from the primary and secondary treatment steps. After collection, the biosolids were air dried to a constant weight. Soil samples were collected from the top 20 cm of an agricultural soil (Skagit silt loam) at the Washington State Research and Extension Center in Mount Vernon, WA. The soil had pH 6.0, organic matter content 22.5 g/kg, and is classified as a silt loam (14.2% sand, 69.8% silt, and 16.0% clay).

**Table 1:** Characteristics of polystyrene nano- and microbeads.

Diameter (*μ*m)	Color	Excitation wavelength Maximum (nm)	Emission wavelength Maximum (nm)	Surface modification	Lot number
0.05	red^a^	542 (green)	612 (red)	none	188495
1.0	blue^a^	365 (UV)	447 (blue)	none	186176
		388 (UV-violet)	447 (blue)		
		412 (violet)	473 (blue)		
2.6	white	na^b^	na^b^	COOH	10050
4.8	green^a^	468 (blue)	508 (green)	none	187677
100	green^a^	468 (blue)	508 (green)	none	185542

All microspheres were from Microgenics Corp., Fremont, CA, except the 2.6 *μ*m were from Bangs Laboratories Inc., IN.

^*a*^ fluorescence color.

^*b*^ na: not applicable.

Permission to sample biosolids was granted by the King County Department of Natural Resources and Parks, Wastewater Treatment Division, Seattle, WA. No permission for soil sampling was required, as the soils were collected from a Washington State University Research Farm. We confirm that the field studies did not involve endangered or protected species.

### Theory of separation of plastic from mineral particles by flotation

Flotation is a common method for separating plastic from mineral solids. A solution with high specific density is used to let plastic particles float to the surface, where the plastic particles are collected. The time for flotation in plastic separation experiments ranges from 20 minutes [13] to 6 hours [14]; however, depending on the size of the plastic particles, the flotation time needs to be adjusted. Plastic particles in the nano- and microsize range may require long flotation times. We can calculate the required flotation time based on the theory of diffusion and sedimentation as outlined below.

The convective velocity *v* of a plastic particle (assuming a spherical shape) in a flotation experiment can be calculated by Stokes’ law:

$$\begin{array}{c} v=\frac{d^{2}a\Delta \rho }{18\eta } \end{array}$$ (1)

where *d* is the diameter of the particle, *a* is acceleration due to gravity or centrifugation, Δ*ρ* is the difference of density between particle and separation fluid, and *η* is the dynamic viscosity. Diffusion of particles can be quantified by the Stokes-Einstein equation *D* = 2*kT*/(6*πηd*), where *D* is the diffusion coefficient, *k* is the Boltzmann constant, and *T* is the absolute temperature. Eq 1 can be used to calculate the time required for flotation if (a) the convective flux dominates the diffusive flux, and (b) if the flow regime is laminar. We consider (a) and (b) valid under the following conditions:

$$\begin{array}{c} \text{Pe}=\frac{vL}{D}\gg 1\;\text{and}\;\text{Re}=\frac{v\rho d}{\eta }\leq 0.1 \end{array}$$ (2)

where Pe is the Peclet number, Re is the Reynolds number, *L* is a characteristic length, taken as the maximum distance of flotation, *ρ* is the density of the fluid, and *v* is the convective velocity given in Eq 1.

**Fig 1:**
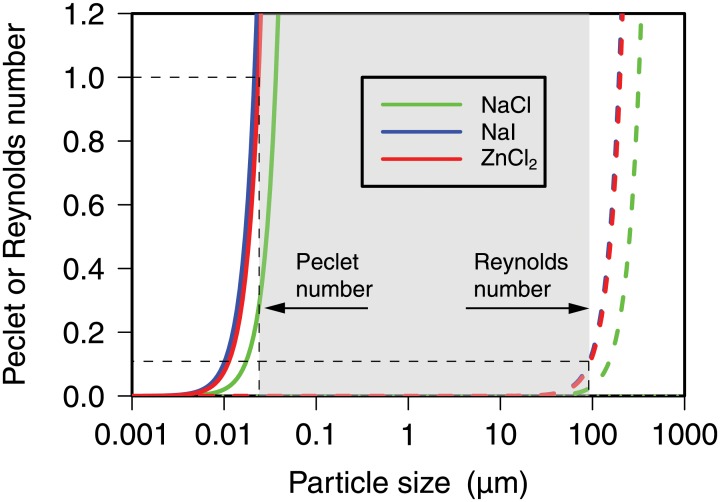
Peclet and Reynolds numbers. Peclet and Reynolds numbers for a spherical particle suspended in different salt solutions as a function of particle diameter. The graph shows pairs of curves of Peclet and Reynolds numbers for three different salt solutions typically used for extraction of plastics: NaCl, ZnCl_2_, and NaI. Curves were calculated using the following parameters (for details on how these parameters were obtained, see Section A in S1 Appendix): *T* = 25°C, *ρ*_NaCl_ = 1.2 g/cm^3^, $\rho_{{\text{ZnCl}}_{2}}=1.6\;\text{g}/{\text{cm}}^{3}$, *ρ*_NaI_ = 1.8 g/cm^3^, *η*_NaCl_ = 1.76 mPa s, $\eta_{{\text{ZnCl}}_{2}}=1.96\;\text{mPa}\;\text{s}$, *η*_NaI_ = 2.32 mPa s. For the density of the plastic particle we assumed *ρ* = 1.05 g/cm^3^ (polystyrene). Shaded ranges show validity of Stokes’ law for the ZnCl_2_ solution.

Fig 1 shows the Peclet and Reynolds numbers as a function of particle diameter, calculated with Eq 2 under normal gravity, for a spherical plastic particle with a specific density of 1.05 g/cm^3^. Peclet and Reynolds numbers were calculated for three salt solutions typically used for plastic extraction. The figure can be used to determine the range of particle sizes for which Stokes’ law is valid. As an example, for ZnCl_2_, this range is from 0.024 to 93 *μ*m, as indicated by the gray box in Fig 1. Particles smaller than 0.024 *μ*m will not separate sufficiently because Brownian motion will prevent them from floating up to the surface. Particles bigger than 93 *μ*m, however, still float up, but not as fast as predicted by Eq 1.

In a typical plastic flotation experiment, soil or sediments and a salt solution are shaken in a beaker. After shaking, heavy soil and sediment particles will settle, while plastics will float. The time *t* for plastic particles to float to the air-water interface can be calculated as:

$$\begin{array}{c} t=\frac{L}{v} \end{array}$$ (3)

where *L* is the maximum distance for particles to reach the air-water interface, and *v* is the velocity given in Eq 1. The efficiency of the plastic extraction can be expressed as:

$$\begin{array}{c} \text{Extraction}\;\text{Efficiency}=\frac{\text{Mass}\;\text{of}\;\text{Plastic}\;\text{at}\;\text{Interface}}{\text{Total}\;\text{Mass}\;\text{of}\;\text{Plastic}} \end{array}$$ (4)

### Experimental verification of separation of plastic from mineral particles by flotation

To test the theory described above, we suspended polystyrene beads in a 100 mL ZnCl_2_ solution (5.64 mol/L, *ρ* = 1.6 g/cm^3^, *η* = 1.96 mPa s). The initial concentrations of the 0.05, 1.0, 2.6, 4.8 and 100 *μ*m polystyrene beads were 50, 50, 100, 100 and 500 mg/L, respectively. We chose different concentrations for the beads so that the number concentrations are not vastly different, but we still could readily measure the beads spectrometrically and gravimetrically.

For the flotation experiments, we found it difficult to precisely sample plastics from the surface of the fluid after flotation in a cylindrical flask or centrifuge tube— the turbulence created during sampling of the plastics with a syringe or vacuum tube made it difficult to quantitatively collect the plastics beads—, so we used a separatory funnel (250 mL Pyrex glass as shown in Fig B in S1 Appendix) that could be drained out without disturbing the plastics collected at or near the air-water interface.

The ZnCl_2_ solution with the polystyrene beads was put into the separatory funnel. At specific times, chosen such that we could construct a time series of the extraction efficiency, we collected the samples by draining the funnels and measuring the top 5% of the volume of the solution for the amount of polystyrene beads. The funnel was rinsed with ZnCl_2_ solution to collect any particles remaining at the funnel walls after the drainage, and the concentration measurements were corrected for the amount of solution added. The amount of polystyrene beads was quantified by UV-Vis spectrometry, except for the 100 *μ*m spheres, which were measured gravimetrically (see details of quantification below).

The theoretical extraction efficiency (Eq 4) in these funnel separation experiments can be calculated as (see Section B in S1 Appendix for details):

$$\begin{array}{c} \text{Extraction}\;\text{Efficiency}=\frac{v(3L^{2}t-3vLt^{2}+v^{2}t^{3})}{L^{3}} \end{array}$$ (5)

where *v* is the flotation velocity (Eq 1), *L* is the maximum distance for flotation, and *t* is the flotation time. The theoretical extraction efficiencies (Eq 5) were then compared with the experimentally measured efficiencies.

### Extraction of plastic from biosolids and soil

#### Spiking biosolids and soil samples with plastic beads

We spiked the biosolids and soil with the polystyrene beads. The spiking was done to obtain mass concentrations of the spheres ranging from 5 to 50 mg/g in the biosolids and 0.5 to 5 mg/g in soil. Given the different size of spheres, the number concentrations varied over several orders of magnitude (Table C in S1 Appendix). For these spiking experiments, we used 1 g of biosolids and 10 g of soil. Biosolids and soil samples were placed into a 250 mL Erlenmeyer flask, and deionized water was added at a biosolid:water ratio (wt:wt) of 5:1 and water:soil ratio of 2:1. Samples were gently shaken by hand to mix the suspension and then let settle for 48 h at ambient temperature. Two types of controls were used: (1) a soil and biosolid blank, i.e. without polystryrene beads, to test for background interference, and (2) polystyrene beads in water only, without soil or biosolids, to test the extraction efficiency under optimal conditions. The controls were subjected to the same treatments as the spiked biosolids and soil samples. These controls should provide 0 and 100% extraction efficiencies, respectively.

#### Removal of natural organic matter by oxidation

We used H_2_O_2_ (30%) to separate the polystyrene beads from natural organic matter in biosolids and soils. The spiked biosolids and soil samples, as well as the controls, were treated by adding 50 mL H_2_O_2_ into the Erlenmeyer flasks. After the initial oxidation reaction had subsided, the flasks were heated with a hotplate to 60°C. As the liquid evaporated and the oxidation reaction continued, we added H_2_O_2_ several times until all of the natural organic matter had oxidized as judged by the cessation of the effervescence and the fading of the dark color. For biosolids 1,600 mL of H_2_O_2_ were needed, for soil 255 mL. For biosolids this treatment lasted 14 days, for soils 7 days. The use of different times for the H_2_O_2_ treatment was because it took much longer for all organic matter to oxidize for the biosolids as compared to the soil. After all organic matter was oxidized, the samples were dried on the hotplate.

#### Separation of plastic beads from biosolids and soil by flotation

To separate the polystyrene beads from the solids remaining after H_2_O_2_ oxidation, 100 mL ZnCl_2_ solution (5.64 mol/L, *ρ* = 1.6 g/cm^3^) was added to the Erlenmeyer flask, which was then sonicated for 20 min, and the suspension then centrifuged for 20 min at 5000 rcf to separate the mineral solids. The supernatant was then transferred into a 250 mL Pyrex glass separatory funnel, and the plastic separated by gravity flotation. The flotation time was chosen to obtain at least 90% extraction efficiency as determined by the theoretical calculations described above. The top 5% of the volume of the suspension was collected for plastic quantification by gravimetry and UV-Vis spectrometry. (Note of caution: high molarity ZnCl_2_ solutions are hazardous, see Section D in S1 Appendix).

#### Extraction of polystyrene beads without H_2_O_2_

We repeated the extraction of the polystyrene beads from biosolids and soils without using H_2_O_2_ to remove the organic matter. The samples were spiked with beads, and then mixed with ZnCl_2_ solution and overhead shaken for 3 min. The solids were let settle and the supernatant transferred to the separatory funnels for the flotation. This procedure did not work with the biosolids as no separation of plastic and the biosolids matrix was possible. So, only results with soils are reported.

#### Effect of H_2_O_2_ oxidation on extraction efficiency of polystyrene beads

To investigate the effect of H_2_O_2_ oxidation on the extraction efficiency of the beads, we exposed the beads to H_2_O_2_ treatment at 60°C for 1 week. Beads were placed into a 250 mL Erlenmeyer flask and 50 mL H_2_O_2_ (30%) was added. Temperature was controlled with a hotplate. After the 1-week exposure, samples were split into two portions: one portion was dried on the hotplate (H_2_O_2_ treated/dried), the other portion was amended with H_2_O_2_ to bring its volume back to 50 mL (H_2_O_2_ treated/not dried). The dried portion was then amended with 50 mL of deionized water and sonicated for 20 min. Then the suspensions were transferred into 250 mL Pyrex glass separatory funnels, and the plastic separated by gravity flotation. The extraction efficiency was measured and calculated by the same method as for the extraction of plastic from biosolids and soil. The concentration of beads in the suspension was quantified by gravimetry and UV-Vis spectrometry. As control, a sample was treated identically but with water instead of H_2_O_2_.

We measured the diameter of the beads under the different tested conditions (control, i.e., beads in water only; H_2_O_2_ treated/not dried; H_2_O_2_ treated/dried). For the 0.05 and 1.0 *μ*m beads, we measured the diameters by laser light scattering (Brookhaven BI-200SM, Brookhaven Instruments Ltd., USA); for the 2.6, 4.8 and 100 *μ*m beads, we measured the diameters with a Malvern laser particle analyzer (Malvern Mastersizer 3000, Malvern Instruments Ltd., UK). We also examined the beads by scanning electron microscopy SEM to determine whether the H_2_O_2_ oxidation would change the surface and shape of the beads.

#### Quantification of polystyrene beads

Polystyrene beads were quantified with UV-Vis spectrometry and gravimetric measurements. For the 0.05, 1.0, 2.6, 4.8 *μ*m beads, we measured the concentrations by absorbance at wavelengths of 216, 316, 290, 480 nm, respectively, with a HP 8452A Diode-Array Spectrometer (Waldbronn, Germany). These wavelengths showed the maximum absorption for the individual bead sizes. Calibration curves for the mass concentrations were developed for each bead size and wavelength. The background solution for the calibrations was the ZnCl_2_ solution. For 100 *μ*m beads, we could not use UV-Vis spectrometry because the beads floated too quickly to the surface; thus we quantified them gravimetrically. Suspensions containing the 100 *μ*m beads were filtered through a 0.45 *μ*m membrane filter (Nitrocellulose membrane, Merck Millipore, Cork, Ireland), air-dried, and the weight determined by an analytical balance after subtraction of the weight of the original filter weight. The measured concentrations were then used to calculate extraction efficiencies (Eq 4).

#### Statistical analysis

All experiments were made with three replicates for each sample. Experimental results for the three replicates were then averaged, and data are reported as means and standard deviations. Statistical analysis was conducted with SPSS 18 for Windows. The extraction efficiencies of microplastic from the different media (biosolids, soil, control) were analyzed with one-way analyses of variance (ANOVA) to test for the effects of medium and particle size on extraction efficiency. Duncan’s multiple range test was performed to test for significance of difference between the treatments (*p* = 0.05).

## Results and discussions

### Flotation time for nano- and microbeads

**Fig 2:**
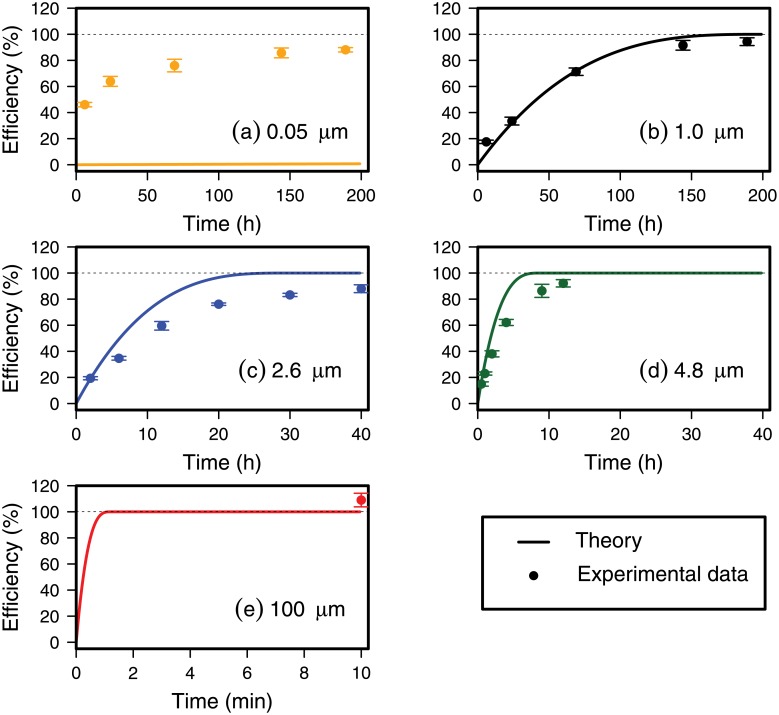
Extraction efficiency for flotation of nano- and microbeads. Extraction efficiency of different size polystyrene beads as a function of time. Beads were suspended on ZnCl_2_ solution in a separatory funnel. Solid lines are theoretical extraction efficiencies calculated with Eq 5, symbols are experimental data, and error bars are ± one standard deviation. Note the different time unit in plot (e).

Extraction efficiencies of polystyrene nano- and microbeads from flotation experiments under normal gravity with a ZnCl_2_ solution are shown in Fig 2. The solid lines show the theoretical extraction efficiencies (Eq 5) as a function of time for different particles sizes. For 0.05 *μ*m particles (Fig 2a), the flotation time is exceedingly long, 100% extraction efficiency is achieved after 86,186 h only. Indeed, the 0.05 *μ*m particle size is close to the lower critical particle size of 0.024 *μ*m particles (Fig 1). As particle size increases to the micrometer-range, the flotation time becomes on the order of 10 to 200 hrs (Fig 2b, 2c and 2d). For particles in the 100 *μ*m range, flotation is fast, in the order of a few minutes (Fig 2e).

The experimentally obtained extraction efficiencies are shown with symbols in Fig 2. For the 0.05 *μ*m beads (Fig 2a) the experimental data did not match the theory, which predicted much longer flotation times to obtain a good extraction efficiency (>90%). We could observe that the beads aggregated, and this would cause the particles to float faster according to their effective aggregation diameter. For the 1.0 *μ*m microspheres (Fig 2b), the experimental data follow the theoretical curves closely. For the 2.6 and 4.8 *μ*m microspheres (Fig 2c and 2d), however, the particles floated less quickly compared to the theory. The 100 *μ*m microspheres floated quickly to the surface of the separatory funnel, much faster than we could sample. Therefore only one sampling point could be measured. According to theory, the extraction efficiency will be 100% after 1.3 min; however, the fastest we could take a sample with our funnel was 10 min, and 109% extraction efficiency was obtained.

Based on our experimental data, 90% extraction efficiency was obtained after 200 h, 200 h, 40 h, 20 h, and 10 min for the 0.05, 1.0, 2.6, 4.8, and 100 *μ*m polystyrene spheres, respectively. These results show that nano- and microplastics can be separated with density separation, but flotation time has to be considered to obtain high extraction efficiencies.

### Extraction efficiency of nano- and microbeads from biosolids and soil

**Fig 3:**
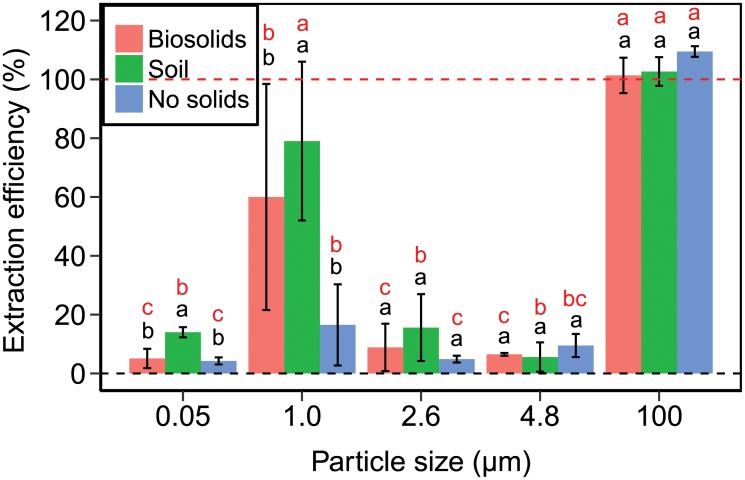
Extraction efficiency of nano- and microbeads from biosolids and soil. Experimental extraction efficiencies of polystyrene beads from biosolids (red columns), soil (green columns), and no solids (blue) by using H_2_O_2_ treatment and flotation. Data represent mean and error bars are ± one standard deviation. The red letters show significant differences of extraction efficiencies among the different size beads, and the black letters show the differences of extraction efficiencies among the different treatments. Extraction efficiencies of control samples without beads were less than 1% (Table E in S1 Appendix), and as such much lower than the measured data shown in the figure.

The experimental extraction efficiencies of polystyrene beads from biosolids and soil are shown in Fig 3. In general, the extraction efficiencies were low: with the exception of the 1 and 100 *μ*m beads, we could recover only about 5 to 16% of the beads. Good recovery (about 100%) was achieved only for the 100 *μ*m beads. The 1 *μ*m beads showed better extraction efficiencies than the 0.05, 2.6, and 4.8 *μ*m beads, but the measurements also showed high standard deviations. We do not have an explanation for this somewhat unexpected result. In most cases, there were no significant differences among the different treatments (biosolids, soil, control). For 0.05 and 1.0 *μ*m beads, the extraction efficiencies from soil were significantly higher than that from biosolids and control. We suspect that the soil minerals protected the beads to some degree from the negative effect of the H_2_O_2_ oxidation. There were significant differences in extraction efficiencies among different bead sizes. Interestingly, the control samples, which did not contain biosolids or soil, also showed low recovery, which is likely caused by the H_2_O_2_; we infer this because flotation in ZnCl_2_ solution without prior oxidation provided high extraction efficiencies (see Fig 2).

### Effect of H_2_O_2_ treatment on nano- and microbeads extraction efficiency

**Fig 4:**
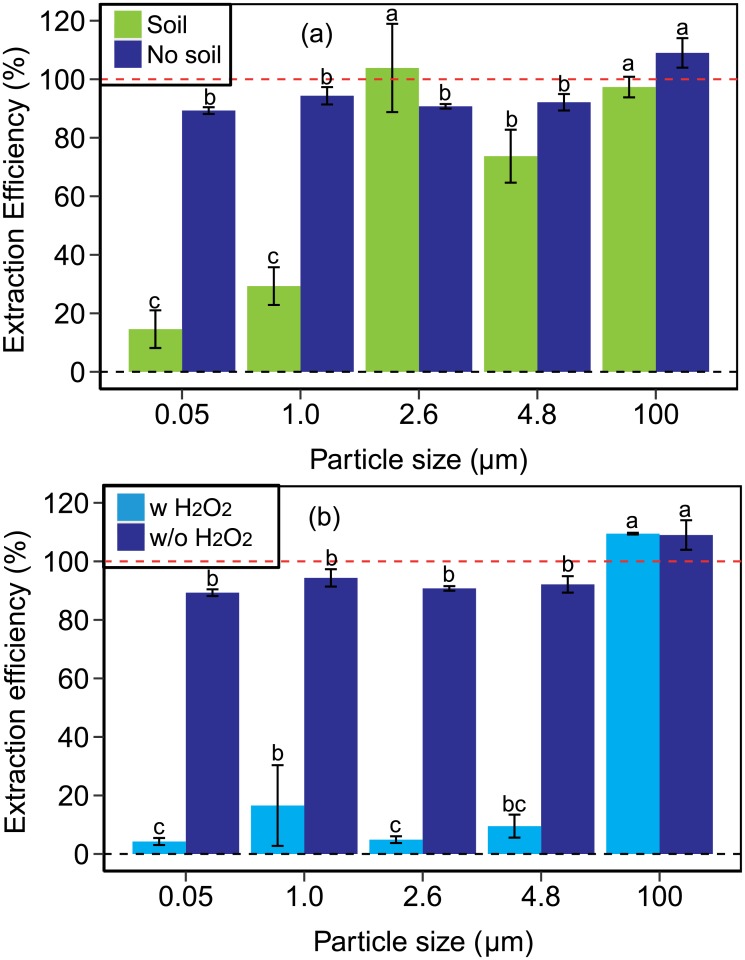
Extraction efficiency for different extraction procedures. Experimental extraction efficiencies of polystyrene beads for different extraction procedures. (a) No H_2_O_2_ digestion, beads were extracted from soil with H_2_O only (no biosolids could be extracted). Extraction efficiencies of control samples without beads were less than 0.5% (Table E in S1 Appendix). The no-soil treatment is extraction from beads in water only, without soil. (b) Beads extracted from water only with and without H_2_O_2_ digestion for 7 days. Note, the dark blue columns in (a) and (b) are the same data. Data represent mean and error bars are ± one standard deviation. Lowercase letters show the differences of extraction efficiencies among different size beads.

To test how the digestion with H_2_O_2_ affected the extraction efficiencies of the polystyrene beads, we repeated the extraction procedure with water only, without using H_2_O_2_. Two treatments were tested: one with soil and water, the other without soil, to serve as control. The extraction with water did not work with the biosolids samples because the large amount of organic matter in the biosolids could not be separated from plastic by flotation. Fig 4a shows that by using water only, the extraction efficiency improved considerably, both for the no-soil treatment (i.e., control) as well as the soil treatment. For the no-soil treatment, reasonable extraction efficiencies (>90%) were obtained. The extraction efficiencies of 100 *μ*m beads were significantly higher than that of the smaller beads, while there were no significant differences among the smaller ones. However, for the soil treatment, the extraction efficiencies were dependent on particle size of the polystyrene beads. For particles ≥2.6 *μ*m, the extraction efficiencies were >74%, but for particles ≤1 *μ*m, the extraction efficiencies dropped to <29%, and were significantly lower than those of the larger beads.

To test whether the H_2_O_2_ treatment affected recovery of beads from water only, we determined the extraction efficiencies with and without H_2_O_2_. No solids (biosolids or soil) were used in this test. Fig 4b shows that in absence of H_2_O_2_ good extraction efficiencies were obtained (>90%); however, with H_2_O_2_, the extraction efficiencies were lower than 17%, except for the 100 *μ*m beads. One of the possible reasons for this lower extraction efficiency is that the oxdidation process caused surface modifications of the beads, particularly of the smaller polystyrene nano- and microbeads (0.05, 1.0, and 2.6 *μ*m) (Section F in S1 Appendix). Data shown in Section F in S1 Appendix suggest that by removing the drying step during the H_2_O_2_ treatment, the polystyrene beads were less affected; nonetheless, the 0.05 and 1.0 *μ*m beads still showed obvious surface modifications.

These results suggest that treatment of soil samples with H_2_O_2_ to remove organic matter negatively affects the extraction of nano- and microplastics from soil, with a more pronounced negative effect on the smaller beads (smaller beads were partially digested by H_2_O_2_, see Section F in S1 Appendix). On the other hand, not using H_2_O_2_ also negatively affects the extraction, but not to such a strong degree. The extraction efficiencies from soil with water only (Fig 4a, left columns) were higher than those with H_2_O_2_ (Fig 3, center columns). We observed that particle size of the plastic was important for the recovery of the beads. While larger plastic beads (≥2.6 *μ*m) could be extracted well from soil with water (Fig 4a, left columns), the smaller beads (≤1.0 *μ*m) could not.

The 100 *μ*m beads could be extracted well from soil, and the extraction efficiencies were not negatively affected by the H_2_O_2_ treatment. This was also true for the extraction of the beads from biosolids. Large plastics thus may be well recovered from biosolids and soils, while plastics in the nano- and lower micrometer size range may not.

## Conclusions

Flotation methods with high density salt solutions are commonly used for extracting microplastics from soils and sediments. High extraction efficiencies are reported in the literature for microplastics in the range of (100 to 250 *μ*m) [27]. However, here we show that for nano- and microplastics in the range of 0.05 to 5 *μ*m, extraction efficiencies from biosolids and soils by density separation are low. While the flotation method works to remove nano- and microplastics from a solution, the extraction from a solid matrix is the limiting step. Removal of organic matter in biosolids and soils with H_2_O_2_ negatively affected extraction efficiencies. Quantification of nano- and microplastics with diameter <5 *μ*m from biosolids and soils is challenging and likely not possible with density separation and flotation.

A reliable extraction method is urgently needed for extraction and subsequent quantification of plastic particles <5 *μ*m in biosolids and soils. This will aid in our ability to characterize potential risk from occurrence and transport of plastics in soil, because this size fraction is the mostly likely to be ingested by soil organisms and the most likely to move through soil pores.

## Supporting information

S1 Appendix Dynamic viscosity of salt solutions; extraction efficiency for a separatory funnel; initial concentrations of polystyrene nano- and microbeads in biosolids and soil; high molar ZnCl_2_ solutions; extraction efficiencies from biosolids and soil control samples without polystyrene nano- and microbeads; effects of H_2_O_2_ treatment on polystyrene beads. (PDF)
