# Supplementary material for: Poor extraction efficiencies of polystyrene nano- and microplastics from biosolids and soil
Source: PLoS One. 2018 Nov 29;13(11):e0208009. doi: 10.1371/journal.pone.0208009 (PMC6264880; doi:10.1371/journal.pone.0208009)
Supplement: S1 Appendix — (PDF) [file pone.0208009.s001.pdf]

S1 Appendix

A Estimation of Dynamic Viscosity of Salt Solutions

For the calculation of Peclet and Reynolds numbers, knowledge of density and viscosity of the salt solutions is required. For salt solutions typically used for plastic flotation, we estimated the dynamic viscosities as a function of fluid density based on literature data and linear interpolation. For viscosities not found in the literature, we estimated the values at a given temperature based on:

$$\eta_1 = \frac{\rho_1}{\rho_2}\eta_2 \tag{A}$$

where η_1 is the dynamic viscosity for a solution with density ρ_1 and η_2 and ρ_2 are a known pair of dynamic viscosity and density of this solution at a given temperature. We deduced this linear relationship between viscosity and density based on data of NaCl reported in [1] (Fig A). Note that Eq A implies that the kinematic viscosity (η/ρ) of a salt solution remains constant when the concentration of the salt solution changes, which may not be always the case, but it appears that this relation holds for NaCl.

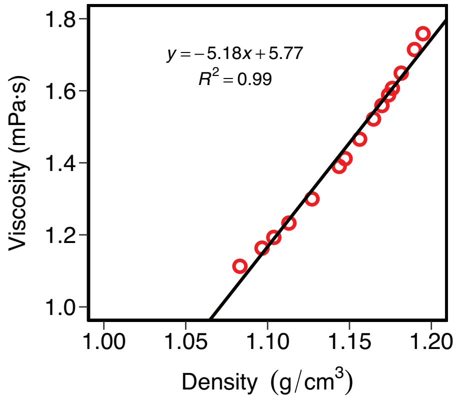

Fig A. Relationship of dynamic viscosity and density of NaCl solutions at 25°C. Experimental data are taken from Zhang and Han [1]. Regression indicates a linear relationship between viscosity and density.

The following table shows densities and dynamic viscosities for NaCl, $ZnCl_2$, and NaI for selected salt concentrations typically used in flotation experiments.

Salt	Concentration	Density (g/cm^3)	Viscosity (mPa s)	T (^{o}C)	Reference
NaCl	6 mol/L	1.19	1.76	25	Zhang and Han [1]
$ZnCl_2$	2 mol/L	1.2	1.47	25	http://www.doc88.com/p-954210904528.html
	6 mol/L	1.6	1.96^b	25	Imhof et al [2], Rocha-Santos and Duarte [3]
NaI	nr^a	1.55	2	20	Palma and Lopes [4]
	nr^a	1.8	2.32^b	25	Nuelle et al [5]

^{a} not reported.
^{b} calculated with Eq A.

B Extraction Efficiency for a Separatory Funnel

For our flotation experiments, we used a separatory funnel. The total mass of plastic particles in the funnel can be calculated as:

$$V_{tot}C_0 = \frac{\pi}{3}\tan^2\alpha\, L^3 C_0 \tag{B}$$

where V_{tot} it the total volume of the suspension used for the extraction, C_0 is the initial particle concentration, L is the height of liquid level in the funnel, and α is the funnel angle (Fig B).

The mass of plastic at the air-water interface can be calculated as the integral of the flux over time multiplied with the cross-section. Because of the funnel shape, we can write this integral as:

$$\int_0^t A(t)\, vC_0\, dt \tag{C}$$

where $A(t) = \pi(L' \tan\alpha)^2$ is the cross-section as a function of flotation time, $L' = vt$ is distance traveled by a particle during time t, v is the Stokes velocity given in Eq 1, and vC_0 is the flux. The extraction efficiency is then given as Eq C divided by Eq B, which yields:

$$\text{Extraction Efficiency} = \frac{v\left(3L^2t - 3vLt^2 + v^2t^3\right)}{L^3} \tag{D}$$

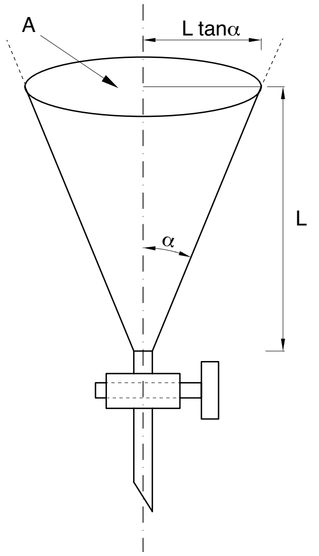

Fig B. Schematic of funnel used for flotation experiments.

C Initial Concentrations of Polystyrene Nano- and Microbeads in Biosolids and Soil

Bead size (μm)	Concentration			
	mass (mg/g)	number (n/g)	mass (mg/g)	number (n/g)
	Biosolids		Soil	
0.05	5	7.2×10^{13}	0.5	7.2×10^{12}
1.0	5	9.0×10^{9}	0.5	9.0×10^{8}
2.6	10	1.0×10^{9}	1	1.0×10^{8}
4.8	10	1.6×10^{8}	1	1.6×10^{7}
100	50	9.0×10^{4}	5	9.0×10^{3}

D High Molar $ZnCl_2$ Solutions

High molarity solution of $ZnCl_2$ have a low pH; a 5.64 mol/L $ZnCl_2$ has a pH = 2. There is also the possibility of precipitation of ZnO nanoparticles out of $ZnCl_2$ when the pH is increased. ZnO nanoparticles are commonly synthesized by titrating $Zn(NO_3)_2$ [6] or $ZnCl_2$ solutions [7]. When a concentrated $ZnCl_2$ is diluted with water, zinc oxychloride particles will precipitate [8,9]. We observed this behavior when we added $ZnCl_2$ solutions to the mixture of water and H_2O_2 with polystyrene beads after oxidation. A white precipitate formed which could be centrifuged out from solution (Fig C).

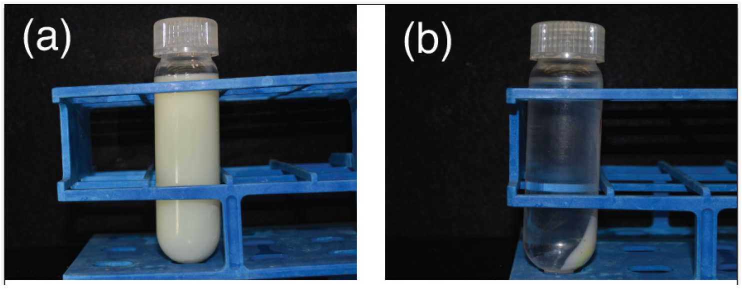

Fig C. Formation of Zn-containing nanoparticles from the mixture of 22 mL $ZnCl_2$ (5.64 mol/L) and 3 mL diluted H_2O_2. (a) Before and (b) after centrifugation.

E Extraction Efficiencies from Biosolids and Soil Control Samples without Polystyrene Nano- and Microbeads

Solid Materials	Bead Diameter			
	0.05 μm	1.0 μm	2.6 μm	4.8 μm
	Extraction Efficiency (%)			
Extraction with H_2O_2				
Biosolids	−1.3	0.3	0.4	0.2
Soil	0.8	−0.3	−0.2	−0.9
Extraction without H_2O_2				
Biosolids	na[†]	na	na	na
Soil	−0.8	0.2	0.4	−0.7

[†]Biosolids could not be extracted without the use of H_2O_2

F Effects of H_2O_2 Treatment on Polystyrene Beads

To investigate the effect of H_2O_2 oxidation on the polystyrene beads, we exposed the beads to H_2O_2 at 60°C for 1 week. Beads were placed into a 250 mL Erlenmeyer flask and 50 mL H_2O_2 (30%) were added. Temperature was controlled with a hotplate. After the 1-week exposure, samples were split into two portions: one portion was dried on the hotplate, the other portion was amended with H_2O_2 to bring its volume back to 50 mL. The dried portion was then amended with 50 mL of deionized water and sonicated for 20 min.

The beads (initial, after H_2O_2 oxidation with or without drying) were then examined by scanning electron microscopy SEM to determine whether the H_2O_2 oxidation would change the surface and shape of the beads.

The SEM images indicate that the 0.05 and 1 μm beads were affected by the H_2O_2 treatment. The surface of these beads became rougher, and the beads became smaller, which is particularly obvious for the 0.05 μm beads that were dried. No obvious alterations were observed for the larger beads (2.6, 4.8, 100 μm).

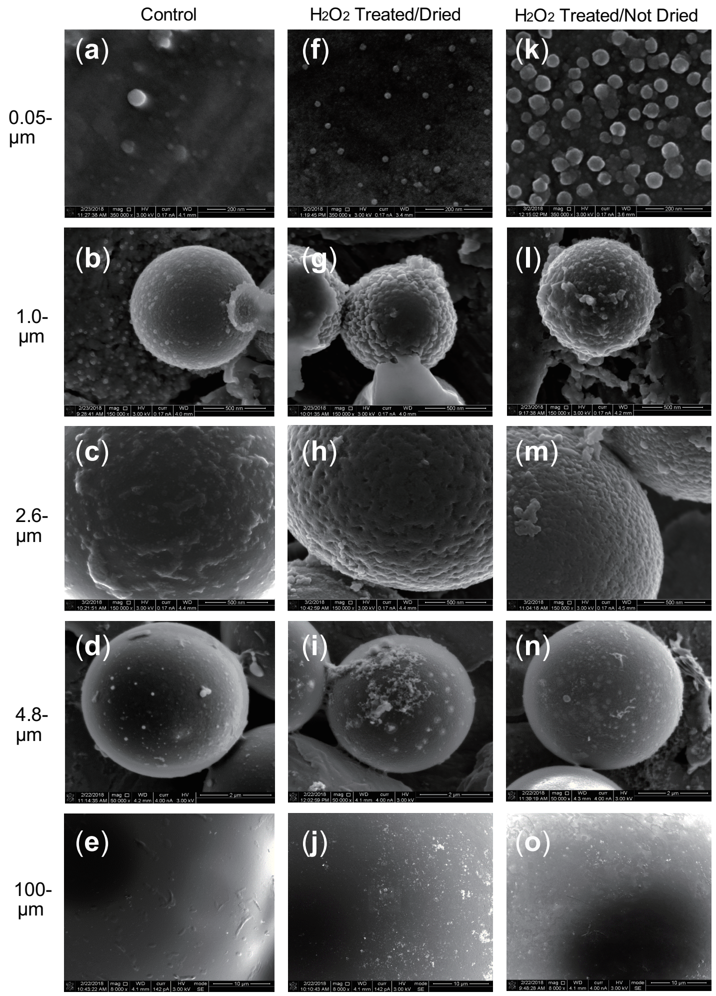

Fig D. Scanning electron micrographs of polystyrene beads: (a–e) untreated; (f–j) treated with 30% H_2O_2 for 7 days and and dried at the end; (k–o) treated with 30% H_2O_2 for 7 days, but not dried.

The beads were also characterized with light scattering. Measured diameters of the beads

are shown in the table below. Smaller beads (0.05 and 1.0 μm) show signs of aggregation after H_2O_2 treatment, indicated by their higher than expected diameter.

	Bead diameter (μm)				
Treatment	0.05	1.0	2.6	4.8	100
	Measured diameter (μm)				
Control	0.06	0.9	2.8	4.8	113
H_2O_2/not dried	6.1	1.7	2.4	4.3	97.6
H_2O_2/dried	8.3	5.3	3.3	5.2	103

References

1. Zhang HL, Han SJ. Viscosity and Density of Water + Sodium Chloride + Potassium Chloride Solutions at 298.15 K. J Chem Eng Data. 1996;41:516–520.
2. Imhof HK, Schmid J, Niessner R, Ivleva NP, Laforsch C. A novel, highly efficient method for the separation and quantification of plastic particles in sediments of aquatic environments. Limnol Oceanogr-Meth. 2012;10:524–537.
3. Santos TR, Duarte AC. A critical overview of the analytical approaches to the occurrence, the fate and the behavior of microplastics in the environment. Trend Anal Chem. 2015;65:47–53.
4. Palma JMLM, Lopes AS. Advances in Turbulence XI, Proceedings of the 11th EUROMECH European Turbulence Conference, June 25-28,2007. Berlin, Germany: Springer; 2007.
5. Nuelle MT, Dekiff JH, Remy D, Fries E. A new analytical approach for monitoring microplastics in marine sediments. Environ Pollut. 2014;184:161–169.
6. Nejati K, Rezvani Z, Pakizevand R. Synthesis of ZnO nanoparticles and investigation of the ionic template effect on their size and shape. Int Nano Lett. 2011;1:75–81.
7. Schmitt M. Synthesis and testing of ZnO nanoparticles for photo-initiation: experimental observation of two different non-migration initiators for bulk polymerization. Nanoscale. 2015;7:9532–9544.
8. Peacock JC, Peacock BLD. Some observations the dissolving of zinc chloride and several suggested solvents. J Pharm Sci. 1918;7:689–697.
9. Holland AC. An investigation into the three component system zinc oxide, hydrochloric acid, zinc chloride and water. University of New Zealand, Christchurch, New Zealand: M.Sc. Thesis; 1928.
